# Authorship of Publications Supported by NCI-Funded Grants Involving Low- and Middle-Income Countries

**DOI:** 10.1001/jamanetworkopen.2024.3215

**Published:** 2024-03-29

**Authors:** Linsey Eldridge, Elise M. Garton, Kalina Duncan, Satish Gopal

**Affiliations:** 1 Center for Global Health, National Cancer Institute, Rockville, Maryland

## Abstract

**Importance:**

Scientific publication is an important tool for knowledge dissemination and career advancement, but authors affiliated with institutions in low- and middle-income countries (LMICs) are historically underrepresented on publications.

**Objective:**

To assess the country income level distribution of author affiliations for publications resulting from National Cancer Institute (NCI)–supported extramural grants between 2015 and 2019, with international collaborating institutions exclusively in 1 or more LMICs.

**Design and Setting:**

This cross-sectional study assessed authorship on publications resulting from NCI-funded grants between October 1, 2015, and September 30, 2019. Grants with collaborators in LMICs were identified in the National Institutes of Health (NIH) Query/View/Report and linked to publications using Dimensions for NIH, published between 2011 and 2020. Statistical analysis was performed from May 2021 to July 2022.

**Main Outcomes and Measures:**

Author institutional affiliation was used to classify author country and related income level as defined by the World Bank. Relative citation ratio and Altmetric data from Dimensions for NIH were used to compare citation impact measures using the Wilcoxon rank sum test.

**Results:**

In this cross-sectional study, 159 grants were awarded to US institutions with collaborators in LMICs, and 5 grants were awarded directly to foreign institutions. These 164 grants resulted in 2428 publications, of which 1242 (51%) did not include any authors affiliated with an institution in an LMIC. In addition, 1884 (78%) and 2009 (83%) publications had a first or last author, respectively, affiliated with a high-income country (HIC). Publications with HIC-affiliated last authors also demonstrated greater citation impact compared with publications with LMIC-affiliated last authors as measured by relative citation ratios and Altmetric Attention Scores; publications with HIC-affiliated first authors also had higher Altmetric Attention Scores.

**Conclusions and Relevance:**

This cross-sectional study suggests that LMIC-affiliated authors were underrepresented on publications resulting from NCI-funded grants involving LMICs. It is critical to promote equitable scientific participation by LMIC institutions in cancer research, including through current and planned programs led by the NCI.

## Key Points

**Question:**

What is the representation of low- and middle-income country (LMIC)–affiliated authors on publications supported by National Cancer Institute (NCI)–funded grants involving LMICs?

**Findings:**

In this cross-sectional study of 164 NCI-funded grants involving LMICs, 51% of all publications did not have any author affiliated with an institution in an LMIC. In addition, 78% and 83% of publications had a first or last author, respectively, affiliated with a high-income country.

**Meaning:**

This study suggests that LMIC-affiliated authors are underrepresented on publications supported by NCI-funded grants involving LMICs.

## Teaser

This cross-sectional study assesses the country income level distribution of author affiliations for publications resulting from National Cancer Institute (NCI)–supported grants between 2015 and 2019, with international collaborating institutions exclusively in 1 or more low- and middle-income countries (LMICs).

## Introduction

Publication of research is an important tool for the dissemination of scientific knowledge that benefits institutions and individuals.^1^ In many settings, authorship is an indicator of scientific contribution, particularly for first and last authors.^2,3^ When researchers from high-income countries (HICs) and low- and middle-income countries (LMICs) collaborate, authorship representation and distribution have been examined as a measure of partnership equity.^4,5^

Previous studies have found underrepresentation of authors affiliated with institutions in LMICs on global health research conducted in LMICs, especially in first and last authorship positions.^6,7,8,9,10,11^ Hedt-Gauthier et al^12^ found that just 53% of first authors of health-related articles in sub-Saharan Africa published between 2014 and 2016 were from the country of the article’s focus and 13.5% had no authors from the country in which the research took place. More recently, Akudinobi and Kilmarx^13^ found an increase in local first and last authors on articles about sub-Saharan Africa funded by the National Institutes of Health (NIH) Fogarty International Center between 2008 and 2020, but by the end of that period, local authors still constituted only 63% and 47% of first and last authors, respectively. The Fogarty International Center focuses much of its funding on capacity building for scientists in LMICs.^14^ Looking at cancer clinical trials specifically, Rubagumya et al^15^ recently found that 33% of oncology clinical trials enrolling participants in LMICs published between 2014 and 2017 did not include a single author from an LMIC.

In 2022, Charani et al^16^ described the inequities that permeate global health research funding, calling on funders to reflect on their processes and offering key metrics toward more equitable global health, including publication equity. The US National Cancer Institute (NCI) is the principal federal agency whose mission is to support and conduct cancer research. The NCI Center for Global Health (CGH) supports the NCI mission by advancing global cancer research and control.^17^ The CGH seeks to specifically support research that addresses key scientific gaps in global cancer control, with a focus on LMICs, where the majority of the global cancer burden occurs and where cancer research efforts have historically been limited.^18^ The CGH also seeks to promote equity in global cancer research collaborations. Building on recent bibliometric studies from noncancer global health disciplines, we sought to describe the authorship and bibliometric characteristics for publications resulting from NCI-supported grants with LMIC collaborators to inform current and future efforts at the NCI and more broadly.

## Methods

### Data Collection

This cross-sectional bibliometric study describes authorship associated with NCI-funded grants. Grants were identified in the NIH internal Query/View/Report system if they were active at any point from October 1, 2015, through September 30, 2019; were administered by the NCI; and exclusively collaborated with institutions in LMICs or were awarded directly to a foreign institution in either an LMIC or an HIC with collaborating sites in LMICs. We assumed that such grants with only LMIC international collaborating sites had LMIC collaboration as a primary research focus and that linked publications should include at least 1 coauthor from an LMIC as an indicator of equitable partnership. We chose to exclude grants with both LMIC and non-LMIC international collaborating sites, assuming that LMIC collaboration may not have been the primary research focus for such grants. Low- and middle-income countries included all countries with World Bank income levels categorized as low income, lower-middle income, and upper-middle income as of 2021. We additionally excluded grants for which only a supplement to the base award was identified as having LMIC collaborators or with the following activity codes: P20 (exploratory grants), P30 (center core grants), and P50 (specialized center). (A supplement is an increase in funding to an existing NIH grant either due to an expansion of the project’s approved scope or research protocol or due to increased costs within the existing scope that were unforeseen when the original funding was awarded.) The latter grant types were excluded because these are large, multiproject awards (eg, NCI-designated cancer center support grants) for which the LMIC component may have been small.^19^ The NIH institutional review board determined that ethical review was not required for this cross-sectional bibliometric study because it was not human participants research.

We linked grants to related publications using Dimensions for NIH, which searches multiple databases for publications with the grant number included in the acknowledgments, funding statement, and PubMed and Crossref listings.^20^ Because grants active from 2015 to 2019 may have been active in years prior, we searched for associated publications between 2011 (the founding year of the NCI Center for Global Health) and 2020 with affiliation information provided for at least 1 author.

Two measures of impact were sourced from Dimensions for NIH: the relative citation ratio (RCR) and the Altmetric Attention Score. The RCR indicates the relative citation performance of an article compared with other articles in its area of research and is calculated for all articles funded by the NIH in the Dimensions for NIH catalog that have been published for at least 2 years and have at least 1 citation.^21^ The Altmetric Attention Score is an algorithm-driven measurement of the attention a publication receives according to 19 source categories, such as social media, patents, and news articles.^22^

### Data Processing

Data cleaning was performed in Microsoft 365, version 2401 (Microsoft Corp) and Python, version 3.0 (Python Software Foundation). Countries were assigned to first and last authors of a publication based on their affiliation listing in Dimensions for NIH. Authors affiliated with institutions in multiple countries were listed as such. Author income class and world region were assigned based on World Bank classification groups in 2021. If first or last authors had multiple affiliations within a single income class (eg, Canada and the US), that income class was counted once. If authors had multiple affiliations across 2 or more income classes (eg, Canada and Ghana), authors were categorized as “dual income classes.” Each publication was assumed to have a single first author and a single last author. Authors of single-author publications were analyzed with first authors. It was not possible to assign specific countries to individual non–first authors or non–last authors, but a list of countries for all authors was used to categorize publications based on the presence or absence of any author from an LMIC.

### Statistical Analysis

Statistical analysis was performed from May 2021 to July 2022. Descriptive statistics were used for authorship position, affiliation, and impact scores. Logistic regression was used to assess changes in authorship over time. The RCR and Altmetric Attention Score data from Dimensions for NIH were used to compare citation impact measures using the Wilcoxon rank sum test. All *P* values were from 2-sided tests and results were deemed statistically significant at *P* < .05. Data analysis was completed in SAS Enterprise Guide, version 7.1 (SAS Institute Inc) and Microsoft 365, version 2401.

## Results

### Grant Characteristics

**Figure 1.**
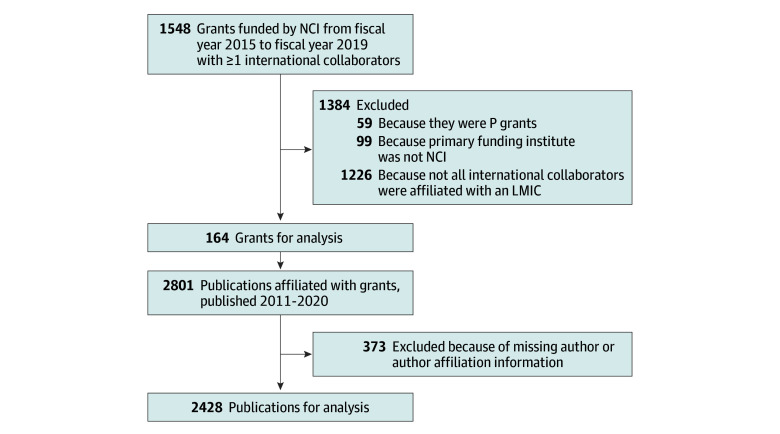
Grant and Publication Selection Program Project/Center Grants (P grants) are large, multiproject efforts that generally include a diverse array of research activities. LMIC indicates low- and middle-income country; NCI, National Cancer Institute.

The initial search resulted in 1548 grants. As shown in Figure 1, 1384 grants were excluded for not meeting the study criteria, resulting in 164 grants for analysis.

**Figure 2.**
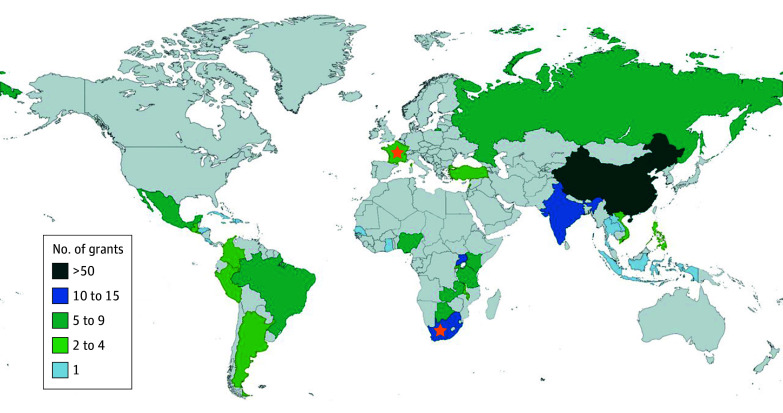
National Cancer Institute–Funded Grants by Country Stars indicate direct grants.

Of these, 159 grants were awarded to US institutions with collaborators in LMICs, and 5 were awarded directly to foreign institutions from 2015 to 2019. Among the 5 direct awards, 3 were awarded to institutions in South Africa, and 2 were awarded to institutions in France with collaborators in Zambia (eTable 1 in Supplement 1). Grants could have more than 1 collaborator from multiple institutions and from multiple countries. US institutions collaborated with 34 LMICs across 6 world regions (Figure 2). Institutions in China collaborated on the greatest number of grants (n = 70), with the second highest being India (n = 14) (eTable 1 in Supplement 1).

### Bibliometric Characteristics

The 164 grants included in this study yielded 2801 publications. After excluding 373 publications with missing author information, the final analysis dataset included 2428 publications (Table 1) between 2011 and 2020. Ten publications (0.4%) with a single author were counted with first authors. Publications with first authors and last authors with dual affiliations from more than 1 income level accounted for 7% of publications each (first authors, n = 174; last authors, n = 168). Detailed characteristics of grants and publications analyzed are shown in Table 1.

**Table 1.** Grant and Publication Characteristics

Characteristic	No. (%)
**Total grants analyzed**	164
Collaboration information	
Direct awards to LMIC institution	3 (2)
Direct awards to HIC institution with LMIC collaborator	2 (1)
Award to US institution with LMIC collaborator	159 (97)
**Total publications analyzed**	2428
Type of publication	
Journal article	2371 (98)
Preprint	28 (1)
Book chapter	18 (0.7)
Conference proceedings	11 (0.5)
Institutional affiliations of first authors	
Affiliated with an LMIC	360 (15)
Affiliated with an HIC	1884 (78)
Affiliated with an LMIC and HIC	174 (7)
Institutional affiliations of last authors	
Affiliated with an LMIC	251 (10)
Affiliated with an HIC	2009 (83)
Affiliated with an LMIC and HIC	168 (7)
Institutional affiliations of any author	
≥1 Author affiliated with an LMIC	1108 (46)
No author affiliated with an LMIC	1242 (51)
≥1 Author affiliated with an LMIC and HIC	168 (7)
Institutional affiliations of first and last authors	
Affiliated with an LMIC	198 (8)
Affiliated with an HIC	1780 (73)
Affiliated with an LMIC and HIC	53 (2)

Abbreviations: HIC, high-income country; LMIC, low- and middle-income country.

Publications included 26 273 total authors with a median of 9 authors (range, 1-113 authors) per publication. These authors were affiliated with institutions in 80 countries, of which 40 were HICs. The number of countries represented among publication authors ranged from 1 (1038 publications) to 29 (2 publications) and are shown in eTable 2 in Supplement 1. The most frequently listed countries of author affiliations on a publication were the US (1750 publications), Canada (362), and the UK (132), with all 10 most frequent country affiliations being HICs apart from China (81 publications).

### Authorship Position

**Figure 3.**
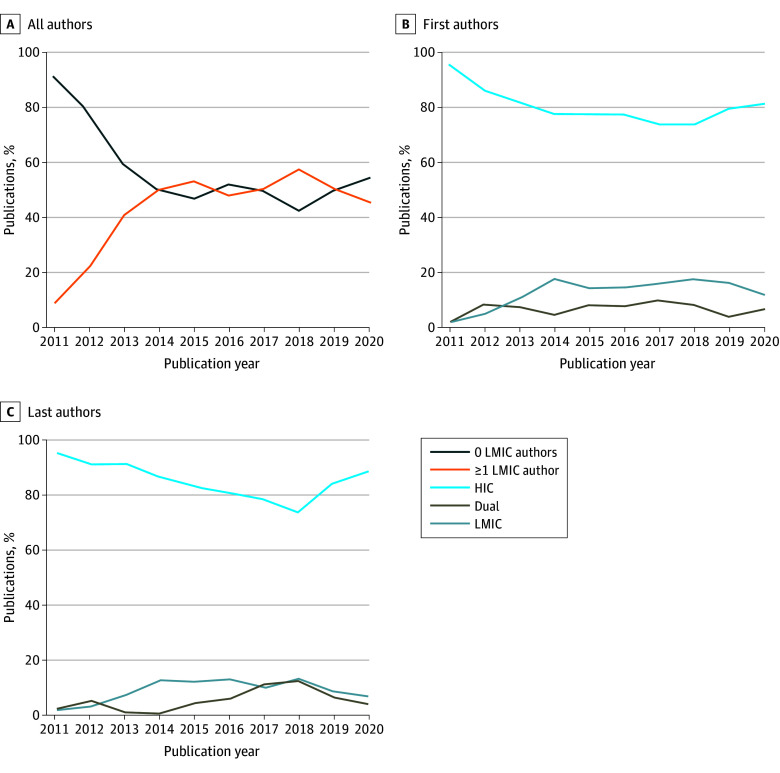
National Cancer Institute–Supported Publication Time Trends by Income Group of Authors’ Country of Affiliation (2011-2020) HIC indicates high-income country; LMIC, low- and middle-income country.

A little over half of all publications did not have any author affiliated with an institution in an LMIC (1242 [51%]). Publications with at least 1 author affiliated with an institution in an LMIC significantly increased from 9% (4 of 44) in 2011 to 46% (178 of 391) in 2020 (*P* = .001). As shown in Figure 3, this percentage gradually increased from 2011 to 2018 and decreased slightly between 2018 and 2020.

Publications with LMIC-affiliated first authors accounted for 15% (n = 360) of all publications. Publications with LMIC-affiliated last authors accounted for 10% (n = 251) of all publications. The proportion of publications with an LMIC-affiliated first or last author did not change significantly over the study period.

Publications with both first and last authors affiliated with institutions in HICs accounted for 73% (n = 1780) of all publications. In addition, 1884 (78%) and 2009 (83%) publications had a first or last author, respectively, affiliated with institutions in HICs. Publications in which the first and last authors were affiliated with institutions in LMICs represented 8% (n = 198) of publications.

The analysis was repeated after removing grants with collaborators exclusively in China (n = 65). No differences were observed in the proportion of publications with LMIC authors when these grants were excluded.

### LMIC-Affiliated First and Last Authors

Publications with an LMIC-affiliated first or last author represented 17% of all publications (n = 413). Among the 360 LMIC-affiliated first author publications, 58% (n = 210) were affiliated with institutions in East Asia and the Pacific, of which 207 were affiliated with institutions in China. Countries in sub-Saharan Africa represented the second-greatest number of LMIC-affiliated first author publications (23% [84 of 360]), with 23 being affiliated with institutions in South Africa.

A similar pattern was seen in LMIC-affiliated last author publications. Among the 251 publications with an LMIC-affiliated last author, 58% (n = 145) were affiliated with institutions in East Asia and the Pacific, and 25% (n = 64) were affiliated with institutions in sub-Saharan Africa. Again, China represented the top affiliated country (n = 140) of all LMIC-affiliated last authors, and South Africa (n = 25) represented the top affiliated country of last authors affiliated with countries in sub-Saharan Africa.

### Publication Impact and Attention

The median RCR for all publications was 1.15 (range, 0.03-213.56). The highest median RCR was found in publications with a dual HIC- and LMIC-affiliated first author (1.18 [range, 0.05-35.36]), and the lowest for LMIC-affiliated last authors (0.92 [range, 0.06-35.52]) (Table 2). The RCR of HIC-affiliated last author publications (1.16 [range, 0.03-213.56]) was significantly higher than for LMIC-affiliated last author publications (0.92 [range, 0.06-35.52]; *P* = .01).

**Table 2.** Bibliometric Output by Country Income of Authors

Authorship position and country income of affiliation	Relative citation ratio		Altmetric Attention Score^a^	
	Median (range)	*P* value	Median (range)	*P* value
First author				
LMIC	1.09 (0.05-35.52)	NA	2 (1-605)	NA
HIC	1.14 (0.03-213.56)	.55	3 (1-1302)	.001
Dual affiliation	1.18 (0.05-35.36)	.51	2 (1-344)	.41
Last author				
LMIC	0.92 (0.06-35.52)	NA	2 (1-90)	NA
HIC	1.16 (0.03-213.56)	.01	3 (1-1302)	<.001
Dual affiliation	1.15 (0.05-35.36)	.09	2 (1-123)	.85

Abbreviations: HIC, high-income country; LMIC, low- and middle-income country; NA, not applicable.

^a^ An algorithm-driven measurement of the attention a publication receives according to 19 source categories, such as social media, patents, and news articles.^22^

The median Altmetric Attention Score for all publications was 3 (range, 1-1302). The highest median Altmetric Attention Score was found in publications with HIC-affiliated first and last authors (3 [range, 1-1302] for each). This median Altmetric Attention Score was significantly higher than for LMIC-affiliated first authors (2 [range, 1-605]; *P* = .001) and LMIC-affiliated last authors (2 [range, 1-90]; *P* < .001) (Table 2).

## Discussion

National Cancer Institute–supported international research is primarily conducted via collaborations between principal investigators at US-based institutions and international collaborators. Of the 7696 grants funded by the NCI in fiscal year 2022, 14% (n = 1054) included an international collaborator and/or principal investigator. Direct awards to international institutions accounted for 48 of those grants, of which 14 were awarded to institutions in LMICs. Partnership is an essential feature of global cancer research and control, which was made even more evident by the COVID-19 pandemic.^23^ Ensuring that partnerships between institutions and investigators in HICs and LMICs are equitable through attention to enabling structural and process issues is critical to advancing impactful, locally relevant global cancer research.

During the 10-year period ending in 2020, despite an overall increase in any LMIC author representation since 2011, more than 50% of publications resulting from NCI-supported grants exclusively with LMIC collaborators did not have a single author affiliated with an LMIC institution, and a low proportion of publications had an LMIC-affiliated first or last author. Publications with HIC-affiliated authors also tended to have higher RCRs and Altmetric Attention Scores, meaning that they received greater attention and were cited more often than publications with LMIC-affiliated authors. Although infectious diseases research is more established in LMICs, our results align with similar bibliometric analyses. Modlin et al^6^ found that of 1308 articles published in prominent infectious diseases journals between 1998 and 2017 with a focus on LIC populations, half had an LIC-affiliated first or last author. Ghani et al^8^ found that LMIC-affiliated authors were reported on just 26% of articles published between 2014 and 2016 across 9 prominent general medicine and global health journals and that more than 28% of all articles reviewed did not include a local author. Tuyishime et al^7^ found that just 45% of first authors and 41% of last authors on publications about sub-Saharan Africa published in *JCO Global Oncology* between 2015 and 2020 were from the region. Regarding bibliometric output, Akudinobi and Kilmarx^13^ reported that the median RCR of publications with first and last authors affiliated with an institution in sub-Saharan Africa was lower than for US-affiliated authors.

Several factors may explain these findings. High-income country–affiliated investigators are often in a more advantageous position to do work associated with first and last authors, such as designing the study, competing for funding, conducting the study, analyzing data, writing the article, and serving as mentors. They are also more likely to be the principal investigator on the award. This places them in a justifiably more prominent publishing position but should also be associated with a responsibility to ensure representation and career development opportunities for their collaborators, including those in LMICs. High-income country–affiliated investigators are more likely to have protected time for research and are often incentivized by their institutions to conduct research and publish articles. These factors may also partly explain the lower Altmetric Attention Scores for publications with LMIC-affiliated authors. High-income country–affiliated investigators, particularly in the US, are also more likely to receive funding for research, even when it is conducted in LMICs.^24^ Finally, perceptions of the importance of authorship may differ across researchers and institutions, which could be a factor in the selection of first and last author positions.^25^ Higher bibliometric scores for publications with HIC affiliations may also be explained by greater institutional resources dedicated to promoting publications and overall greater scientific output from HICs, resulting in greater citations of past publications.^26^

Despite these challenges, our results demonstrate some progress with respect to the proportion over time of authors affiliated with institutions in LMICs. The NCI, the NIH, and the global oncology community have attempted to further this progress in several ways. As one example, the American Society of Clinical Oncology Academic Global Oncology Task Force published 13 recommendations in 2020 to advance equitable global cancer research and control.^27^ Recommendations were organized by training, research and practice, professional development, and collaboration, specifically promoting equitable collaborations between high- and low- or middle-resource settings, such as by recognizing LMIC contributions.

There have also been recent efforts to build capacity for global cancer research in LMICs as part of cancer research programs, such as the NCI Global Training for Research and Equity in Cancer (GlobTREC) program. GlobTREC supports global research training and facilitates building research environments to conduct locally relevant cancer research in LMICs, in part by using Fogarty International Center research training models that have decades-long track records of success.^28,29^ The program requires that at least 50% of all trainees recruited be from LMIC institutions. Another example is the Beginning Investigator Grant for Catalytic Research (BIG Cat) program, originally launched in 2010 by the CGH in partnership with the African Organization for Research and Training in Cancer (AORTIC), to support cancer research conducted by early-career investigators from African countries.^30^ These types of research and training investments seek to support partnerships that embody global health equity principles, including mutual respect and benefit, trust, good communication, and clear partner roles and expectations.^31^

Structural adjustments by funders and academic journals to improve authorship equity can also play a role. In 2021, the CGH changed the criteria for accepted scientific abstracts at the Annual Symposium on Global Cancer Research, held in collaboration with many HIC and LMIC partners, to ensure that research conducted in or about an LMIC includes a coauthor affiliated with that country. The wider academic global oncology community has also recently worked to address inequities in authorship. The oncology journal *ecancermedicalscience* announced in 2020 that it would only accept submissions with at least 1 author from an LMIC or with a significant impact of underresourced settings, citing the imbalance in global scientific publishing and the lack of available data for policymaking and cancer planning in LMICs.^32^ Similarly, *The Lancet* stated in 2022 that it would not accept manuscripts that contain data from African countries without acknowledgment of collaborators from African countries.^33^ In early 2023, *Nature* removed open access publishing fees for authors from more than 70 LMICs.^34^

### Limitations

There are limitations to this bibliometric analysis. First, authorship is a limited measurement of equity and may not be perceived equally across institutions, countries, and cultures.^26,35,36^ Similarly, the traditional model of ascribing authorship is increasingly being challenged as collaborative research and team science grows.^37^ Second, Dimensions for NIH links grants and publications based on grant information provided by the author or grantee, which may be inflated,^38^ leading to inclusion of publications not relevant to the work conducted in collaboration with the LMIC collaborator listed on the grant. This may also explain why publications did not include an LMIC-affiliated author. Third, we assigned author country by their institutional affiliation, which does not account for changing affiliations throughout an author’s career or for the scientific diaspora. In addition, institution information was missing for 13% of publications. Fourth, recent actions to address authorship inequities noted may have resulted in improvements in authorship representation not captured in this analysis, which examined articles only published prior to 2020. Conversely, the effects of the COVID-19 pandemic on research capacity and output may have exacerbated existing authorship disparities.^39^

## Conclusions

This cross-sectional study of publications supported by NCI grants awarded from fiscal year 2015 to fiscal year 2019 directly to LMICs or exclusively with LMIC collaborators revealed that the percentage of LMIC-affiliated first and last authors remained low. The percentage of publications with at least 1 author affiliated with an LMIC increased but remained below 50%. Furthermore, bibliometric output was lower for publications with LMIC-affiliated authors than for those with HIC-affiliated authors. In 2022, funders were asked to reflect on their global health research funding processes, including by supporting representation of LMIC authors on research outputs.^16^ These findings will provide an important benchmark when assessing strategies to promote equitable scientific participation by LMIC institutions and investigators in global cancer research, including through many current and planned NCI programs. This will be particularly important as the share of global cancer burden borne by LMICs continues to increase in coming decades.

## Notes

Supplement 1. **eTable 1.** NCI-Funded Grants by Country and Region **eTable 2.** Countries Represented by Author Affiliation

Supplement 2.
Data Sharing Statement
