# Supplementary material for: Authorship of Publications Supported by NCI-Funded Grants Involving Low- and Middle-Income Countries
Source: JAMA Netw Open. 2024 Mar 29;7(3):e243215. doi: 10.1001/jamanetworkopen.2024.3215 (PMC10980966; doi:10.1001/jamanetworkopen.2024.3215)
Supplement: Supplement 1. — eTable 1. NCI-Funded Grants by Country and Region eTable 2. Countries Represented by Author Affiliation [file jamanetwopen-e243215-s001.pdf]

Supplementary Online Content

Eldridge L, Garton EM, Duncan K, Gopal S. Authorship of publications supported by NCI-funded grants involving low- and middle-income countries. *JAMA Netw Open.* 2024;7(3):e243215. doi:10.1001/jamanetworkopen.2024.3215

eTable 1. NCI-Funded Grants by Country and Region

eTable 2. Countries Represented by Author Affiliation

This supplementary material has been provided by the authors to give readers additional information about their work.

© 2024 Eldridge L et al. *JAMA Network Open.*

eTable 1. NCI-Funded Grants by Country and Region

Country of institution	Indirect grants (n=159)	Direct grants (n=5)	Total grants (n=164)
**East Asia & Pacific**			
China	70		70
Indonesia	1		1
Laos	1		1
Philippines	2		2
Thailand	1		1
Vietnam	2		2
**Europe & Central Asia**			
France[a]		2	2
Russia	9		9
Turkey	2		2
**Latin America & Caribbean**			
Argentina	4		4
Brazil	7		7
Colombia	3		3
Costa Rica	1		1
Cuba	1		1
Dominican Republic	1		1
El Salvador	3		3
Guatemala	3		3
Haiti	1		1
Honduras	1		1
Jamaica	1		1
Mexico	6		6
Peru	4		4
**Middle East & North Africa**			
Lebanon	2		2
**South Asia**			
India	14		14
**Sub-Saharan Africa**			
Botswana	5		5
Ghana	1		1
Kenya	8		8
Malawi	4		4
Nigeria	5		5
Rwanda	4		4
Senegal	1		1
South Africa	8	3	11
Tanzania	9		9
Uganda	13		13
Zambia	4	2	6

[a] Two grants were awarded to institutions in France with collaborators in Zambia.

© 2024 Eldridge L et al. *JAMA Network Open.*

eTable 2. Countries Represented by Author Affiliation

Country of author institution	Number of publications with at least one author institution in country No. (%)
**United States**	1750(72.1)
**Canada**	362(14.9)
**United Kingdom**	132(5.4)
**Netherlands**	116(4.8)
**France**	99(4.1)
**Australia**	97(4.0)
**Germany**	96(4.0)
**Italy**	88(3.6)
**Switzerland**	84(3.5)
**China**	81(3.3)
**Belgium**	66(2.7)
**Japan**	53(2.2)
**Spain**	50(2.1)
**Israel**	48(2.0)
**Sweden**	36(1.5)
**Egypt**	24(1.0)
**Greece**	23(0.9)
**Austria**	22(0.9)
**Denmark**	22(0.9)
**Taiwan**	20(0.8)
**India**	20(0.8)
**Singapore**	18(0.7)
**Brazil**	17(0.7)
**Kenya**	16(0.7)
**South Korea**	15(0.6)
**Tanzania**	14(0.6)
**Hungary**	14(0.6)
**Czechia**	12(0.5)
**Poland**	10(0.4)
**Norway**	10(0.4)
**Morocco**	10(0.4)
**Portugal**	9(0.4)
**Finland**	9(0.4)
**Russia**	7(0.3)
**South Africa**	7(0.3)
**Ireland**	6(0.2)
**Tunisia**	6(0.2)
**Turkey**	6(0.2)
**Peru**	6(0.2)
**Slovenia**	5(0.2)

© 2024 Eldridge L et al. *JAMA Network Open.*

**Colombia**	5(0.2)
**Latvia**	5(0.2)
**Lebanon**	5(0.2)
**Cyprus**	4(0.2)
**Pakistan**	4(0.2)
**New Zealand**	4(0.2)
**Qatar**	4(0.2)
**Iceland**	4(0.2)
**Lithuania**	4(0.2)
**Ukraine**	3(0.1)
**Chile**	3(0.1)
**United Arab Emirates**	3(0.1)
**Mexico**	3(0.1)
**Iran**	2(0.1)
**Jamaica**	2(0.1)
**Belarus**	2(0.1)
**Guatemala**	2(0.1)
**Thailand**	2(0.1)
**Saudi Arabia**	2(0.1)
**North Macedonia**	2(0.1)
**Ghana**	2(0.1)
**Argentina**	2(0.1)
**Vietnam**	1(<0.1)
**Jordan**	1(<0.1)
**Burkina Faso**	1(<0.1)
**Nigeria**	1(<0.1)
**Niger**	1(<0.1)
**Albania**	1(<0.1)
**Philippines**	1(<0.1)
**Zambia**	1(<0.1)
**Bulgaria**	1(<0.1)
**Costa Rica**	1(<0.1)
**Haiti**	1(<0.1)
**Serbia**	1(<0.1)
**Kuwait**	1(<0.1)
**Panama**	1(<0.1)
**Malaysia**	1(<0.1)
**Antigua and Barbuda**	1(<0.1)
**Luxembourg**	1(<0.1)
**Uganda**	1(<0.1)
**Zimbabwe**	1(<0.1)

© 2024 Eldridge L et al. *JAMA Network Open.*
