# Supplementary material for: Authorship of Publications Supported by NCI-Funded Grants Involving Low- and Middle-Income Countries
Source: JAMA Netw Open. 2024 Mar 29;7(3):e243215. doi: 10.1001/jamanetworkopen.2024.3215 (PMC10980966; doi:10.1001/jamanetworkopen.2024.3215)
Supplement: Supplement 2. — Data Sharing Statement [file jamanetwopen-e243215-s002.pdf]

Data Sharing Statement

Eldridge. Authorship of Publications Supported by NCI-Funded Grants Involving Low- and Middle-Income Countries. *JAMA Netw Open*. Published March 29, 2024. doi:10.1001/jamanetworkopen.2024.3215

Data
Data available: No

Additional Information
Explanation for why data not available: The data that support the findings of this study are openly available in NIH World Report.
